# Reproductive outcome of hysteroscopic metroplasty for women with T-shaped uterus: a retrospective study

**DOI:** 10.1186/s12978-022-01381-2

**Published:** 2022-03-28

**Authors:** Yuxin Zhang, Yiping Zhu, Beilei Ge, Mengsong Sui, Zhenzhen Zheng, Jing Sun

**Affiliations:** Department of Reproductive Surgery, Shanghai First Maternity and Infant Hospital, School of Medicine, Tongji University, Shanghai, 200092 China

**Keywords:** T-shaped uterus, Infertility, Hysteroscopic metroplasty, Reproductive outcome

## Abstract

**Background:**

T-shaped uterus is a Müllerian malformation with unapparent clinical manifestations. Intrauterine adhesion and tuberculosis may lead to T-shaped uterus, too. Hysteroscopic metroplasty is a treatment option for T-shaped uterus, while the postoperative reproductive outcomes have not been thoroughly investigated. The aim of this study was to determine the reproductive outcome in Chinese women with T-shaped uterus who had hysteroscopic metroplasty with cold scissors.

**Methods:**

This retrospective cohort study was conducted in the reproductive surgery unit of a university-affiliated hospital. One hundred and eleven patients with T-shaped uterus who underwent hysteroscopic metroplasty from Jan. 2017 to Sept. 2019 were followed-up by telephone in Apr. 2021. All patients received hysteroscopic metroplasty using microcissors, followed by estrogen-progesterone sequential treatment, with or without intrauterine device (IUD) implantation. According to whether they had had history of intrauterine operation, patients were divided into congenital group and acquired group. The main outcome measure was postoperative live birth rate. *χ*^2^ test and *t* test were used for comparison between groups. Cochran-Mantel–Haenszel test were used for stratified analysis. *P* < 0.05 was considered statistically significant.

**Results:**

One hundred and eleven patients were included in total, with 46 in congenital group and 65 in acquired group. After hysteroscopic metroplasty, in the congenital group, the pregnancy rate increased from 28.3% to 87.0% (*P* < 0.001) and the live birth rate increased from 23.1% to 79.5% (*P* = 0.001); in the acquired group, the pregnancy rate slightly dropped from 98.5% to 72.3% (*P* < 0.001) while the live birth rate increased from 20.8% to 74.5% (*P* < 0.001). No statistically significant difference was observed in postoperative reproductive outcome indicators between the two subgroups except mode of conception.

**Conclusions:**

For both groups, hysteroscopic metroplasty may improve reproductive outcomes for patients with T-shaped uterus.

**Supplementary Information:**

The online version contains supplementary material available at 10.1186/s12978-022-01381-2.

## Plain language summary

As a Müllerian malformation, T-shaped uterus is named for the shape of the uterine cavity. According to cause of the disease and patients’ intrauterine operation history, T-shaped uterus can be divided into congenital and acquired types.

This study was conducted in the reproductive surgery unit in a university-affiliated hospital. Data were collected from medical records, and patients were followed up via telephone.

One hundred and eleven patients were included in this study, with 46 in congenital group and 65 in acquired group. Whether infertility/subfertility patients had had intrauterine operation history or not, their rates of giving live birth increased after the hysteroscopic metroplasty.

In conclusion, hysteroscopic metroplasty is an effective intervention for T-shaped uterus patients with fertility intention.

**Supplementary Information:**

The online version contains supplementary material available at 10.1186/s12978-022-01381-2.

## Background

T-shaped uterus is a Müllerian malformation with the uterine cavity shape similar to the letter T. In other cases, it may be secondary to intrauterine adhesion or tuberculosis [1, 2]. There are no clear diagnostic criteria for T-shaped uterus so far [1]. European Society of Human Reproduction and Embryology (ESHRE) and European Society for Gynaecological Endoscopy (ESGE) classification system of female genital anomalies defined it as a narrow uterine cavity due to thickened lateral walls with a correlation of 2/3 uterine corpus and 1/3 cervix [3].

T-shaped uterus was first reported as a diethylstilbestrol (DES) -exposure-related condition by Kaufman et al. in 1977. With the use of DES being banned for pregnant women, the T-shaped uterus still occurs with some unknown mechanism. The reported prevalence of T-shaped uterus varies from 0.2 to 10%[4]. The change of uterine cavity shape and volume may contribute to infertility [5, 6].

Most T-shaped uterus patients have normal menstrual cycles, leaving them not diagnosed until seeking care for infertility or subfertility. Hysteroscopic metroplasty is a treatment choice for these patients [7], but its safety and effectiveness are scarcely reported. There have been several studies focusing on hysteroscopic metroplasty for patients with T-shaped uterus and analyzing postoperative reproductive outcomes. Their live birth rates varied from 43% to 87.5% [7–17]. However, most studies had a limited sample size [7–15] and used monopolar or bipolar [7, 9–11, 14–16] systems instead of cold scissors during the surgery. Besides, Data for East Asia have not been reported so far.

In previous diagnostic hysterolaparoscopies, we have found plenty of T-shaped uterus cases. Some of those patients had no history of intrauterine operation but had been unproperly classified as intrauterine adhesion. We aim to determine the reproductive outcome in Chinese women with T-shaped uterus who had hysteroscopic metroplasty with non-electric instruments. The objectives of this study are 1. To compare the preoperative and postoperative reproductive outcomes. 2. To compare postoperative reproductive outcomes between congenital and acquired T-shaped uterus.

## Materials and methods

### Study design and setting

This retrospective cohort study was conducted at Shanghai First Maternity and Infant Hospital, School of Medicine, Tongji University.

### Patients

In this retrospective study, medical records of inpatient hysteroscopy from Jan. 2017 to Sept. 2019 were searched. Inclusion criteria were (1) women who were diagnosed with T-shaped uterus by hysteroscopy and received subsequent metroplasty and (2) unable to conceive after one year (or longer) of unprotected sex or repeated implantation failure (RIF). Exclusion criteria were (1) history of hysteroscopic metroplasty in other hospitals or units; (2) no fertility intention; (3) severe systemic disease; (4) other gynecological condition that may cause infertility, including submucosal leiomyoma, ovarian endometriosis, intrauterine adhesion except T-shaped uterus, septate uterus, and endometrial polyp; (5) malignancy; (6) fail to undergo a second hysteroscopy.

Considering the T-shaped uterus can occur secondary to intrauterine adhesion[1, 13], all patients included in the study were divided into 2 groups according to their medical history[13]: congenital T-shaped uterus group, patients without a history of intrauterine operation, and acquired T-shaped uterus group, patients with a history of intrauterine operation, e.g. dilation and curettage.

### Clinical procedure

All patients received pelvic ultrasonography at the outpatient department. All patients' medical history, including operation history and childbearing history, was taken upon admission. Blood and urine routine tests, blood biochemical tests, coagulation tests, and electrocardiogram were completed preoperatively to rule out surgical contraindications.

**Fig. 1:**
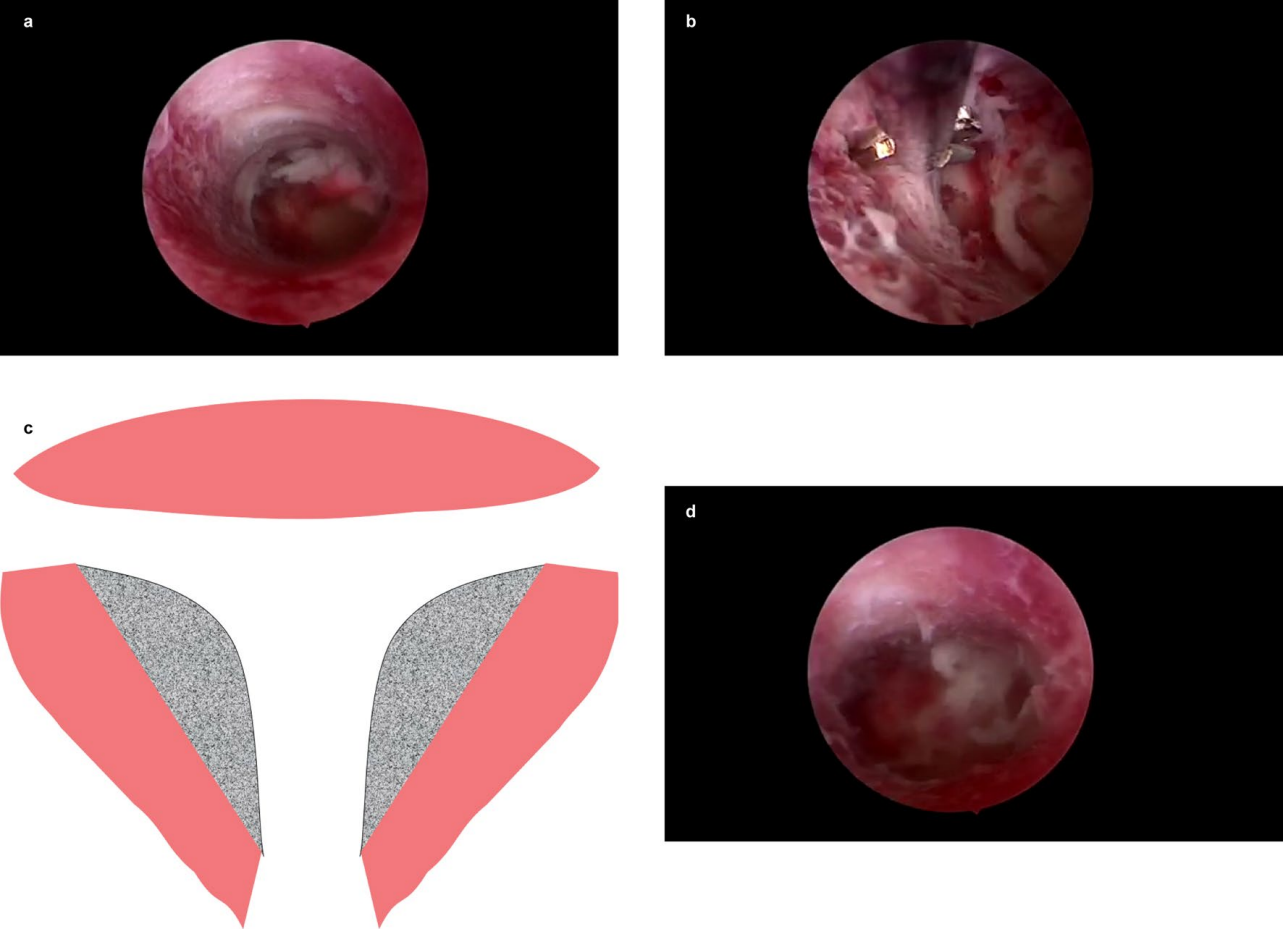
Hysteroscopic metroplasty for a T-shaped uterine cavity. **a**, **b** and **d** are screencaptures of the Additional file 1. **a** Uterine cavity before metroplasty. **b** Longitudinal incision by microscissors on the lateral side under hysteroscopy. **c** Gray area indicates incised area during the operation. **d** Normal shape of uterine cavity was restored after hysteroscopic metroplasty

At proliferative phase of the menstrual cycle (day 3–7 after menses phase), diagnostic hysteroscopy was performed in lithotomy position under intravenous anesthesia. After vulvovaginal disinfection and draping, uterine position was checked by bimanual examination. The cervix was dilated to Hegar 4.5–7 and uterine depth was measured by hysterometer. A 6-mm continuous-flow hysteroscope fitted with scissors (HAWK, Hangzhou, China) was used. After exhausting air, the 0.9% NaCl solution was applied as distention medium with irrigation pressure at 120 mmHg. The scope successively entered ectocervix, endocervical canal, and the endocervix. Uterine cavity, both tubal ostia, the condition of endometrium, and the ratio between endocervical canal length and uterine cavity depth were examined to rule out other lesions. T-shaped uterus was diagnosed according to the ESHRE/ESGE consensus on the classification of female genital tract congenital anomalies [3] by a skilled gynecologist under hysteroscope, which is a narrow uterine cavity due to thickened lateral walls involving 2/3 uterine corpus and 1/3 cervix with both tubal ostia invisible (Fig. 1a). longitudinal incisions were made on both lateral sides with microscissors (Fig. 1b, c) to enlarge the uterine cavity, restoring its normal shape with both tubal ostia uncovered (Fig. 1d and Additional file 1). An inert copper intrauterine device (IUD) was then implanted to prevent intrauterine adhesion. To build an artificial menstrual cycle, patients were prescribed 2 mg oral estradiol valerate b.i.d. from postoperative day 1 to day 21 and 5 mg megestrol acetate q.d. from day 17 to day 21 for 2–3 cycles. Then a second hysteroscopy was scheduled to remove the IUD and reexamine the shape of uterine cavity, followed by another 1–2 artificial menstrual cycles. After that, they were allowed for conception attempt. For patients (1) whose uterine cavity was enlarged less than 10% after metroplasty, (2) with urgent fertility intention, or (3) who refused IUD implantation, the IUD was not implanted. They could try to conceive after finishing artificial menstrual cycles without second hysteroscopy.

### Data collection

Demographic characteristics were acquired in the electronic medical record system of Shanghai First Maternity and Infant Hospital. Obstetrical data were collected by telephone interview. When interviewed by telephone, Subjects were provided with the purpose of the study, and they may refuse to participate in the study without giving any reason. Hypomenorrhea was defined as less than 30 mL bleeding per menstrual cycle. Clinical pregnancy was defined as amenorrhea, positive urine/blood pregnancy test, and intrauterine pregnancy diagnosed by ultrasonography. Spontaneous miscarriage was defined as spontaneous loss of a fetus before 28 weeks of pregnancy according to Chinese expert consensus [18]; live birth was defined as delivery of a fetus with any sign of life after exiting the maternal body beyond 20 weeks of gestation. Follow-up was conducted in Apr. 2021 (at least 18 months after the final hysteroscopy).

### Statistical analysis

Pregnancy rate was defined as the proportion of pregnant patients to all patients. Spontaneous miscarriage rate was defined as the proportion of spontaneous miscarriages to total pregnancies while induced abortion numbers weren’t included; live birth rate was defined as the proportion of live births to total pregnancies with ongoing pregnancies excluded. Biochemical pregnancies weren't included due to the limitation of the retrospective study design.

Statistical analyses were performed using SPSS 24.0 (IBM, Armonk, NY). Measurement data are presented as mean ± SD and categorical data were presented as number (percentage). Measurement data were compared by two independent-sample *t* test while categorical variables were compared by *χ*^*2*^ test. Stratified analyses were performed by Cochran-Mantel–Haenszel (CMH) test. Statistical differences were considered significant when *P* value < 0.05.

## Results

**Fig. 2:**
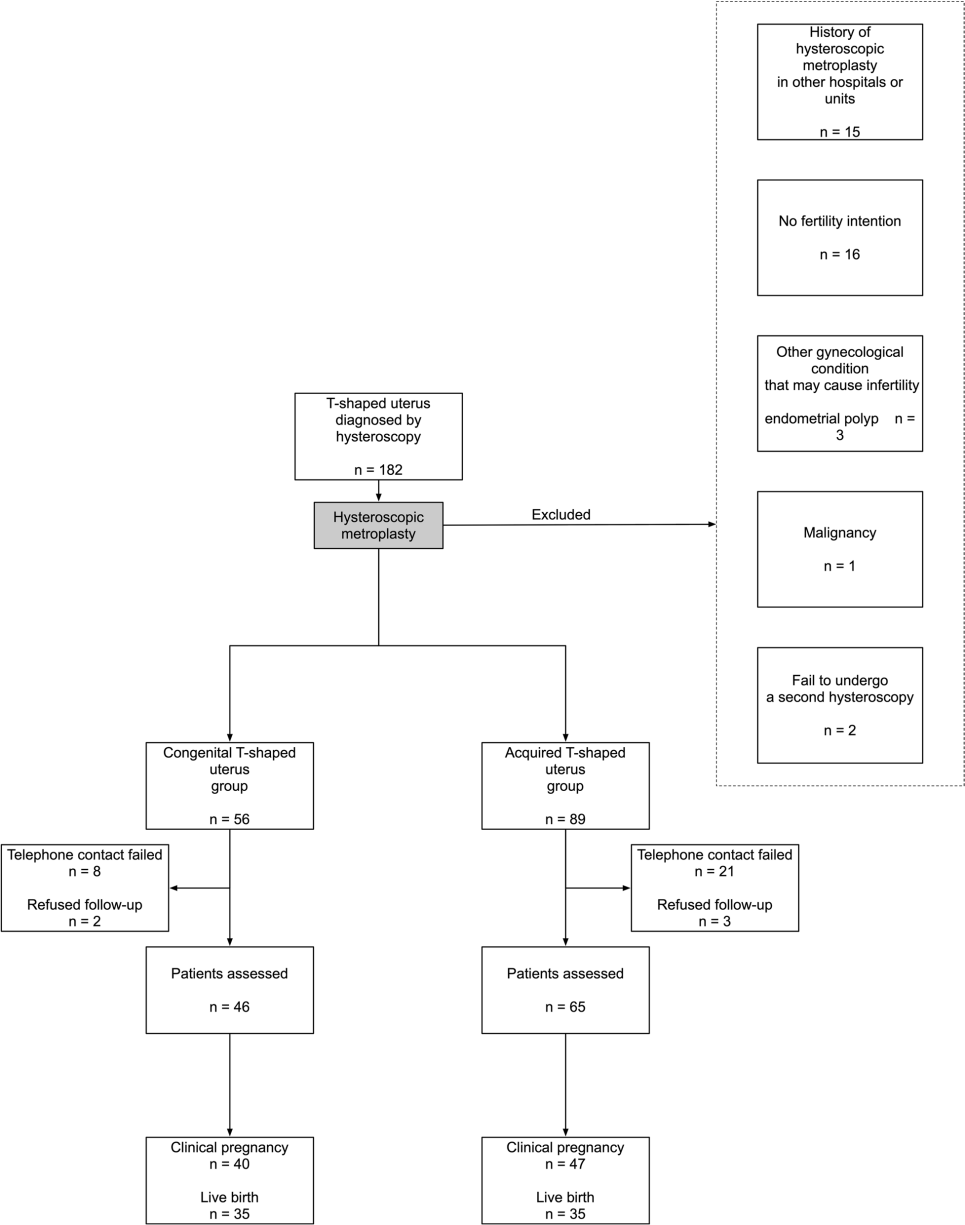
Flow chart of the study design

One hundred and eighty-two subjects were diagnosed with T-shaped uterus by hysteroscopy, 37 of whom were excluded according to the criteria. Eventually, telephone surveys of 111 subjects, who were aged from 22 to 44 years, were completed (Fig. 2). All hysteroscopic metroplasties were performed successfully without complication.

**Fig. 3:**
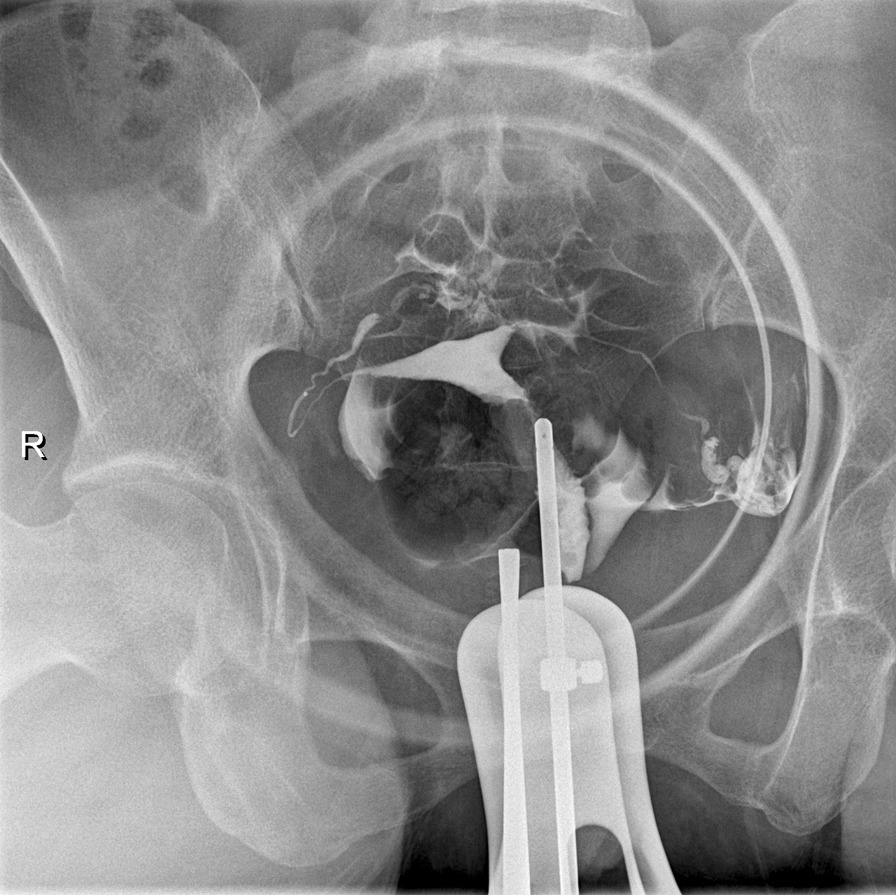
Hysterosalpingogram of one patient included. The left side of the uterine cavity is unconventionally narrowed

Baseline characteristics of included subjects are summarized in Table 1. Age of the two groups (31.4 ± 3.8 years vs. 33.7 ± 4.4 years) had statistically significant difference (*P* < 0.05). Acquired group had higher pregnancy rate (*P* < 0.001), while the pregnancy outcomes between two groups were alike. Hypomenorrhea rate in acquired group was higher as well. More patients in acquired group had their endometrial anomaly detected by preoperative imaging (*P* < 0.001), either by hysterosalpingography (HSG) (Fig. 3) or ultrasonography. Rates of patients with additional infertility factors in two groups were similar (80.4% vs. 86.2%, *P* = 0.618), with difference of constituent ratio of additional infertility factors statistically significant (*P* = 0.006).

**Table 1:** Compaing preoperative demographic and clinical characteristics of the patients

	Congenital group	Acquired group	Total	Test of significance	*P* value
n	46 (41.4%)	65 (58.6%)	111		
Age (yrs)	31.4 ± 3.8	33.7 ± 4.4	32.7 ± 4.3	*t* = 2.839	0.005^*^
BMI (kg/m^2^)	22.0 ± 3.5	21.9 ± 2.9	21.9 ± 3.2	*t* = 0.068	0.946
Previously pregnant patients	13 (28.3%)	64 (98.5%)	77 (69.4%)	*χ*^2^ = 62.475	< 0.001^*^
Previous pregnancies	13	132	145		
Spontaneous miscarriage	9 (69.2%)	57 (43.2%)	66	*χ*^2^ = 3.238	0.072
Live birth	3 (23.1%)	15 (11.4%)	18	*χ*^2^ = 0.610^a^	0.435
Hypomenorrhea	4 (8.7%)	28 (43.1%)	32	*χ*^2^ = 15.519	< 0.001^*^
Imaging diagnosis	8	35		*χ*^2^ = 15.084	< 0.001^*^
HSG	5 (62.5%)	12 (34.3%)			
Ultrasonography	3 (37.5%)	20 (57.1%)			
HSG & ultrasonography	0	3 (8.6%)			
Infertility duration (month)	30.7 ± 19.7	31.5 ± 29.7		*χ*^2^ = 0.179	0.858
Patients with additional infertility factors	37 (80.4%)	56 (86.2%)		*χ*^2^ = 12.262^b^	0.006*^c^
Ovulatory	1	0			
Tubal	20	20			
Unilateral	3	3			
Bilateral	17	17			
Male	0	1			
Age	2	18			
Multiple	14	17			

*P < 0.05, ^a^Yates's correction for continuity, ^b^Fisher's exact test, ^c^Comparing constituent ratio

During the surgery, 3 (4.6%) patients in acquired group were observed with endometrial congestion, but no patient in congenital group was observed. There were 14 (30.4%) patients in congenital group and 10 (15.4%) received hysteroscopy combined with laparoscopy. The difference of combined laparoscopy rates was not statistically significant. (*P* = 0.066).

Hysteroscopic metroplasty improved live birth rate and decreased spontaneous miscarriage rate greatly in both groups (Table 2a, b). Pre- and postoperative live birth rates were 23.1% vs. 79.5% (*P* = 0.001) in congenital group and 20.8% vs. 74.5% (*P* < 0.001) in acquired group. Spontaneous miscarriage rates were 69.2% vs.20.5% and 79.2% vs. 25.5% respectively. Pregnancy rate in congenital group increased from 28.3% to 87.0% (*P* < 0.001) after hysteroscopic metroplasty. In acquired group, pregnancy rate dropped from 98.5% to 72.3% (*P* < 0.001). All postoperative ongoing pregnancies (1 vs. 5) in the two groups were in the second or third trimester.

**Table 2:** **a** Comparing preoperative with postoperative pregnancy outcomes in congenital group. **b** Comparing preoperative with postoperative pregnancy outcomes in acquired group

	Preoperative	Postoperative	*χ*^2^	*P* value
a				
n	46			
Pregnancy patients	13 (28.3%)	40 (87.0%)	32.447	< 0.001^*^
Pregnancy outcome^a^	n = 12	n = 44		
Live birth	3 (23.1%)	35 (79.5%)	10.482	0.001^*b^
Spontaneous miscarriage	9 (69.2%)	9 (20.5%)		
b				
n	65			
Pregnancy patients	64 (98.5%)	47 (72.3%)	17.814	< 0.001^*^
Pregnancy outcome^a^	n = 72	n = 47		
Live birth	15 (20.8%)	35 (74.5%)	33.578	< 0.001^*^
Spontaneous miscarriage	57 (79.2%)	12 (25.5%)		

*P < 0.05 ^a^Ongoing pregnancies excluded ^b^Yate's correction for continuity

Postoperative reproductive outcomes were also compared between the two groups (Table 3). There was no significant difference in the pregnancy rates between the two groups (*P* = 0.065). The main type of conception differed between the two groups (*P* = 0.027), i.e. congenital group conceived mainly with assisted reproductive technology (ART) while more patients in acquired group conceived spontaneously. But for patients with live birth, the difference of their conception mode was not significant between the two groups (*P* = 0.467). The live birth rate was slightly higher in congenital group (77.8%) than in the acquired group (67.3%), and spontaneous miscarriage rate was lower in congenital group (20.0%) than in acquired group (23.1%). But the difference of pregnancy outcomes was not statistically significant (*P* = 0.566). The mode of delivery between the two groups had no statistical difference (*P* = 0.806). As fertility declines as age advances, we compared postoperative reproductive outcomes between patients aged ≤ 35 years old and those > 35 years old. However, neither congenital or acquired group revealed age-related statistical difference in reproductive outcomes (Table 4).

**Table 3:** Postoperative reproductive outcomes of congenital and acquired T-shaped uterus group

	Congenital group n = 46	Acquired group n = 65	*χ*^2^	*P* value
Pregnancy patients	40 (87.0%)	47 (72.3%)	3.411	0.065
Mode of conception^a^	n = 45	n = 52		
Spontaneous conception	15 (33.3%)	29 (55.8%)	4.899	0.027*
ART	30 (66.7%)	23 (44.2%)		
Pregnancy outcome^b^	n = 44	n = 47		
Spontaneous miscarriage	9 (20.0%)	12 (23.1%)	0.330	0.566
Live birth	35 (77.8%)	35 (67.3%)		
Conception mode of live birth	n = 35	n = 35		
Spontaneous conception	13 (37.1%)	16 (45.7%)	0.530	0.467
ART	22 (62.9%)	19 (54.3%)		
Mode of delivery^c^	n = 35	n = 35		
Cesarean section	22 (62.9%)	21 (60.0%)	0.060	0.806
Vaginal birth	13 (37.1%)	14 (40.0%)		

^a^Ongoing pregnancy included, ^b^Ongoing pregnancy excluded, ^c^Total number of live births, ^*^P < 0.05

**Table 4:** **a** Comparing postoperative reproductive outcomes between different age subgroups in congenital group. **b** Comparing postoperative reproductive outcomes between different age subgroups in acquired group

	≤ 35 yrs	> 35 yrs	*χ*^2^	*P* value
a				
n	41	5		
Pregnancy patients	35 (85.3%)	5 (100.0%)	/	1.000^a^
Mode of conception	n = 40	n = 5		
Spontaneous conception	15 (37.5%)	0	1.378	0.24^b^
ART	25 (62.5%)	5 (100.0%)		
Pregnancy outcome^c^	n = 39	n = 5		
Live birth	31 (79.5%)	4 (80.0%)	0	1.000^b^
Spontaneous miscarriage	8 (20.5%)	1 (20.0%)		
Conception mode of live birth	n = 31	n = 4		
Spontaneous conception	13 (41.9%)	0	/	0.274^a^
ART	18 (58.1%)	4 (100.0%)		
b				
n	43	22		
Pregnancy patients	34 (79.1%)	13 (59.1%)	2.901	0.089
Mode of conception	n = 38	n = 14		
Spontaneous conception	21 (55.3%)	8 (57.1%)	0.015	0.904
ART	17 (44.7%)	6 (42.9%)		
Pregnancy outcome^c^	n = 33	n = 14		
Live birth	26 (78.8%)	9 (64.3%)	0.458	0.498^b^
Spontaneous miscarriage	7 (21.1%)	5 (35.7%)		
Conception mode of live birth	n = 26	n = 9		
Spontaneous conception	12 (46.2%)	4 (44.4%)	/	1.000^a^
ART	14 (53.8%)	5 (55.6%)		

^a^Fisher's exact test, ^b^Yate's correction for continuity, ^c^Ongoing pregnancies excluded

Given the noticeable proportions of patients not receiving IUD in both groups, whether receiving IUD intraoperatively could be a potential confounding covariate. CMH test was applied for stratified analysis [19]. All Breslow-Day tests [20] were insignificant (*P* > 0.05), indicating that odds ratios between IUD and non-IUD patients had no statistically significant difference (Table 5). Whether patients received IUD or not, reproductive outcome ameliorations were consistent in both congenital and acquired group.

**Table 5:** Outcomes of CMH statistics

	Odds ratio	95% CI		*P* value	*P* value^†^
		Lower	Upper		
Congenital group					
Pregnancy patients	15.154	5.332	43.069	< 0.001*	0.100
Live birth	37.741	10.324	137.968	< 0.001*	0.110
Acquired group					
Pregnancy patients	0.044	0.006	0.328	0.002*	0.214
Live birth	3.921	1.835	8.378	< 0.001*	0.630

*P < 0.05

^†^Significance of Breslow-Day test for homogeneity

## Discussion

The study evaluated reproductive outcomes of hysteroscopic metroplasty for women with T-shaped uterus. The impact of T-shaped uterus on reproduction is not determined yet [1, 4]. In this study, all included patients suffered infertility or recurrent miscarriage before surgical correction. The preoperative mean infertility duration in congenital and acquired group were 30.7 months and 31.5 months, respectively. After the hysteroscopic metroplasty, 87.0% of patients in the congenital group and 72.3% of patients in the acquired group conceived successfully. When compared with preoperative counterparts, patients' live birth rates increased and spontaneous miscarriage rates dropped significantly, regardless of cause of the disease. Together with other similar studies [7–17], our study confirms that hysteroscopic metroplasty is an effective option to restore fertility for women with T-shaped uterus. The treatment mechanisms may involve restoring normal uterine cavity [21] and change of endometrial receptivity [22].

After DES was banned from use during pregnancy, causes of T-shaped uterus still remains unclear. Intrauterine adhesion and genital tuberculosis could be risk factors of T-shaped uterus [2]. Since intrauterine adhesions may occur in women with tuberculosis [7] but no included patient in this study had had tuberculosis before, we divided patients into those two groups according to possible cause of T-shaped uterus. Comparison of postoperative reproduction outcome between congenital and acquired group has not been reported yet. We found significant increase in live birth rates and significant decrease in spontaneous miscarriage rates in both groups. Whether patients have history of intrauterine operation or not, hysteroscopic metroplasty may improve reproductive outcomes for women with T-shaped uterus. Interestingly, for pregnancies, more patients in congenital group conceived with ART while more patients in acquired group conceived spontaneously (*P* < 0.05). But for live births, the mode of conception had no significant difference between the two groups (*P* > 0.05). In this study, mode of conception was recommended on the basis of their individual fertility status. After the metroplasty, gynecologists should thoroughly evaluate condition of fallopian tubes and other combined infertility factors of the patient to give her proper advice about conception in the future.

The mean age of the patients in our study is similar to those in the literature [13, 16, 23]. The live birth rates in our study were greatly higher than most of the studies included in a recent meta-analysis [24]. Considering the number of ongoing pregnancies and loss to follow-up, the eventual pregnancy rate should even be higher. We did not take age limit into inclusion criteria of this study. Stratified comparison on age demonstrated no statistical significance in reproductive outcomes, suggesting that the main cause for infertility in this study may lie in factors other than ovarian ones. Even so, this conclusion requires confirmation with larger sample size. Given fertility decline with age [25, 26], we still recommend patients with fertility intention to try and perceive as soon as possible.

Our study also showed that whether receiving IUD intraoperatively is not a confounding factor when comparing pre- and postoperative reproductive outcomes. However, the IUD implantation decision in our study was not completely based on subjective manifestations and could be affected by patient’s desire. The impact of IUD implantation should be investigated thoroughly by high-quality randomized controlled trials (RCTs) in the future.

During the procedure, cold microscissors were used as cutting instrument. We recommend cold scissors for patients with fertility intention, which may prevent collateral thermal damage [27, 28] to endometrium. For studies used electrosurgery systems in large samples [7, 9, 15, 17], pregnancy rates varied from 49.5% to 66.1%, live birth rates varied from 63.2% to 86.7%, and miscarriage rates varied from 11.9% to 28.1%.While the overall pregnancy rate, overall live birth rate, and overall miscarriage rate in our study was 78.4%, 76.9%, and 23.1%, respectively. The pregnancy outcomes were similar but our study had a slightly higher pregnancy rate. Alonso Pacheco et al. [13] reported a pregnancy rate of 83.3% in a similar study which avoided electrosurgery. The highest live birth rate was reported by Garbin et al. in 1998, which was 87.5%, but the sample size was small (n = 24). They used monopolar hook during the surgery. Therefore, we may speculate that cold scissors in hysteroscopic metroplasty for T-shaped uterus can lead to a higher pregnancy rate than electrosurgical systems, but live birth rates and spontaneous miscarriage rates are alike. Since pregnancy count is the denominator when calculating live birth rate and spontaneous miscarriage rate, the raise of pregnancy rate may still indicate some advantages of cold scissors. Considering various possible confounding factors, whether thermal damage caused by electrosurgery systems have negative effect on patients' long-term fertility remains to be explored by comparative studies in the future.

Though additional infertility factors had different compositions between the two groups, patients with indications for ART were all referred to reproductive medicine specialists, which eases the damage of additional fertility factors to reproductive outcomes. However, not all patients in this study have received comprehensive examination for infertility factors. Detailed tests for additional infertility factors followed by targeted treatment could benefit infertile patients more.

This study has some limitations, too. Firstly, the diagnosis of T-shaped uterus in this study was based on surgeon's subjective judgment under hysteroscopic observation. There are no clear diagnostic criteria for T-shaped uterus [1]. We found that neither HSG or ultrasonography can detect endometrial anomaly effectively, especially for patients without intrauterine operation history. Radiologists tend to concentrate more on tubal patency and the ratio between endocervical canal length and uterine cavity depth in this study, the shape of uterine cavity could be occasionally ignored. T-shaped uterus were diagnosed by experienced gynecologists under hysteroscopic observation, which may result in missed diagnosis for those with atypical manifestations. As the imaging technology develops, the use of 3D ultrasonography is getting more and more popular. 3D ultrasound could measure the lateral wall thickness and cavity depth of the uterus precisely [29], making it possible to develop objective and standardized diagnostic criteria. Alonso Pacheco et al. [30] have proposed an IYT classification system based on transvaginal 3D ultrasound for T-shaped uterus, whose value will be confirmed in the future. In hospitals where 3D ultrasonography is not available, gynecologists should be cautious about the uterine cavity shape in hysteroscopy. Secondly, this study did not set a true control group for ethical reasons. A true control group here should contain contemporary patients with T-shaped uterus who did not receive hysteroscopic metroplasty. Though there was no data about reproductive outcomes of true control group, the comparison of pre- and postoperative figures may indicate the effectiveness of hysteroscopic metroplasty in a way. Thirdly, this retrospective study design restricted our access to some information such as biochemical pregnancies. And telephone interview may lead to loss of follow-up and recall errors. Despite these shortcomings, this study provides data on Chinese women and analyzed patients with T-shaped uterus according to possible causes for the first time. Large prospective, multicenter study may deliver more solid evidence in the future.

## Conclusion

Given the improved reproductive outcomes in our study and previous researches, hysteroscopic metroplasty is an effective intervention for T-shaped uterus patients with fertility intention. For both congenital and acquired group, hysteroscopic metroplasty with cold knife may improve reproductive outcomes for women with T-shaped uterus.

## Supplementary Information

**Additional file 1.** Video of hysteroscopic metroplasty.

## Data Availability

The datasets used and analysed during the current study are available from the corresponding author on reasonable request.

## Abbreviations

IUD: Intrauterine device
ESHRE: European Society of Human Reproduction and Embryology
ESGE: European Society for Gynaecological Endoscopy
DES: Diethylstilbestrol
RIF: Repeated implantation failure
CMH test: Cochran-Mantel–Haenszel test
HSG: Hysterosalpingography
ART: Assisted reproductive technology
RCT: Randomised controlled trials
